# Consuming Different Structural Parts of Bamboo Induce Gut Microbiome Changes in Captive Giant Pandas

**DOI:** 10.1007/s00284-021-02503-y

**Published:** 2021-06-09

**Authors:** Zheng Yan, Qin Xu, Walter H. Hsu, Stephan Schmitz Esser, James Ayala, Rong Hou, Ying Yao, Dandan Jiang, Shibin Yuan, Hairui Wang

**Affiliations:** 1 Chengdu Research Base of Giant Panda Breeding, Chengdu, 610081 China; 2 Sichuan Key Laboratory of Conservation Biology for Endangered Wildlife, Chengdu, 610081 China; 3 Sichuan Academy of Giant Panda, Chengdu, 610081 China; 4 Key Laboratory of Southwest China Wildlife Resources Conservation of Ministry of Education, Nanchong, 637009 China; 5 Department of Biomedical Sciences, Iowa State University, Ames, IA 50011 USA; 6 Department of Animal Science, Iowa State University, Ames, IA 50011 USA

## Abstract

Giant pandas consume different structural parts of bamboo (shoots, leaves and culms) during different seasons. Previous research showed different bamboo parts have varying nutritional content and that a long-term diet consisting of a single part of bamboo resulted in remarkable metabolic changes within captive giant pandas. However, the effects on the gut microbiome of giant pandas, as a result of a single bamboo part diet, have not been investigated. Here, we evaluated the changes in gut microbial communities based on single bamboo part diets and their potential implications by using 16S rRNA gene-based amplicon sequencing and metagenome shotgun sequencing. We found that the composition and function of the gut microbiome from captive giant pandas fed exclusively culms were significantly different from that of individuals fed shoots or leaves. During the culm feeding period, the gut microbiome showed strongest digestive capabilities for cellulose, hemicellulose and starch, and had the highest potential abilities for the biosynthesis of bile acids, fatty acids and amino acids. This suggests the microbiome aids in breaking down culm, which is more difficult for giant pandas to digest, as a means to compensate for the nutrient poor content of the culm. Genes related to fatty acid metabolism and tricarboxylic acid cycle enzymes were more abundant during the leaf stage diet than that in the shoot and culm stages. Thus, the microbiome may help giant pandas, which typically have low lipase levels, with fat digestion. These results illustrate that adaptive changes in the gut microbiome community and function may be an important mechanism to aid giant panda digestion when consuming different structural parts of bamboo.

**Supplementary Information:**

The online version contains supplementary material available at 10.1007/s00284-021-02503-y.

## Introduction

The giant panda (*Ailuropoda melanoleuca*) is a member of the Order Carnivora with > 99% of its diet composed of bamboo. Up to 14 h each day, giant pandas consume 10–40 kg of fresh bamboo (10–16 kg leaves/culms or 30–40 kg shoots) [1]. However, the giant panda still retains the typical digestive tract of a carnivore. The short intestinal tract of the giant panda, evolved for meat consumption, causes vegetative matter to be expelled quickly without full digestion [2]. In addition, the lower lipase activity [3] and the lack of enzyme genes for cellulose digestion [4] lead to the panda's own limited digestion of food. Moreover, the protein content of bamboo is low whereas the fiber and lignin content level are high [5–7]. Hence, how giant pandas maintain their own nutritional requirements is a topic of interest. Previous research showed that giant pandas have a low daily energy expenditure [8]. Moreover, metagenomic research revealed that the gut microbiota of giant pandas may play a vital role in bamboo digestion, increasing absorption and transformation of nutrients [9, 10]. Recent studies have shown that compared with other omnivorous or herbivorous animals, the abundance of putative cellulolytic genes from giant pandas is low, while α-amylase and hemicellulase gene families are high [11, 12].

Giant pandas browse on different structural parts of several species of bamboo during different seasons, with shoots primarily consumed in spring and summer, leaves primarily in autumn and winter, and culms in late winter and early spring [13]. Studies on nutrition, ingestion and utilization of bamboo show that the protein, fat, sugar, fiber and cyanide level vary markedly in different bamboo plant parts [5, 6, 13–15]. In a previous study, we found significant differences at the nutrient level in the different parts of bamboo: shoots are rich in crude protein and culms contain abundant crude fiber; while both leaves and shoots contain ample crude fat [5]. The apparent digestibility of bamboo parts from giant panda’s diet is also different [5], which results in marked differences in the hindgut’s nutrient components used by gut microbes. In addition, long-term consumption of a single bamboo part can lead to significant metabolic changes in captive giant pandas [5]. However, possible changes of the composition and function of the giant panda’s gut microbiota when consuming different bamboo parts are still unknown. To address this issue, we investigated changes in the gut microbiomes of captive giant pandas when provisioned exclusively with a diet consisting of either bamboo shoots, leaves or culms using the 16S rRNA gene and metagenome shotgun sequencing techniques.

## Materials and Methods

### Subjects and Animal Provisioning

A total of 19 adult captive giant pandas (11 number male, 8 number female), ranging from 8–17 years of age (average age was 11), were the subjects for the current study. All subjects were housed at the Chengdu Research Base of Giant Panda Breeding (CRBGPB), located in Chengdu, Sichuan province, PRC and were considered healthy and did not require any medical treatments during the study, including antibiotics, which might affect sample analysis. In addition, no subject was pregnant or lactating during this experiment. All subjects were singly housed and fed according to the normal husbandry practices of the CRBGPB with bamboo and water provided ad libitum. Dietary supplements were provided by body mass with the same proportion between individuals. At the CRBGPB, giant pandas are provisioned with bamboo that is seasonally available, usually with bamboo shoots in autumn, bamboo leaves in winter and bamboo culm in early spring. Thus, three study groups consisting of nine mixed sexed giant pandas were formed based on the bamboo parts provided. The bamboo species provided were *Phyllostachys bissetii* (culm group), *Bashania fargesii* (leaf group) and *Qiongzhuea opienenss* (shoot group), from which giant pandas only eat the culm, leaves and shoots, respectively.

### Collection of Fecal Samples

For each of the three study groups, nine fecal samples were collected from each of the nine subjects after the corresponding bamboo species/structural parts were continuously provided for at least 20 days (Table S1 and Fig. S1). In total, 27 fecal samples from 19 pandas were collected from October 2016 to June 2017. Fecal samples were collected within 10 min of defecation. The outer layer, which was in contact with the ground, was removed to avoid contamination of the sample and the remainder was packaged and stored at -80 ºC for further analysis.

### DNA Extraction

A pretreatment before DNA extraction was performed using the methods described in Xue et al. [16]. Fecal DNA was extracted from pretreated cell suspension using the QIAamp^®^ DNA Stool Mini Kit (Qiagen, Germany) following the manufacturer’s specifications. DNA concentration and purity were monitored on 1% agarose gels. The final DNA concentration of each sample was used for subsequent 16S rRNA and metagenome shotgun.

### 16S rRNA Gene Amplification, Sequencing and Bioinformatics Analysis

The extracted DNA was amplified using the specific primer (16S V4: 515F—806R) with the corresponding barcode. Sequencing libraries were constructed using the TruSeq® DNA PCR-Free Sample Preparation Kit (Illumina, USA) according to the manufacturer’s instruction. Subsequently, the constructed library quality was evaluated on a Qubit@ 2.0 Fluorometer (Thermo Scientific) and an Agilent Bioanalyzer 2100 system prior to sequencing. Lastly, the library was sequenced on an Illumina HiSeq 2500 provided by Novogene Biological Information Technology Co (Beijing, China) and 250 bp paired-end reads were achieved.

Paired-end reads were merged and filtered using FLASH (V1.2.7) [17] and Quantitative Insights Into Microbial Ecology (QIIME) software package Version 1.9.1 to obtain the high-quality clean tags [18]. Chimera sequences within the clean tags were removed using the UCHIME Algorithm program to obtain clean data (Effective Tags) [19]. Clean data with ≥ 97% similarity were classified as the same Operational Taxonomic Unit (OTU) by Uparse software (v7.0.1001) [20].We selected a representative sequence of each OTU for the taxonomic annotation using the RDP classifier Version 2.2 [21] and the GreenGene Database (http://greengenes.lbl.gov/cgi-bin/nph-index.cgi) [22]. The Alpha diversity, Rarefaction Curve, Rank Abundance Curve and Principal Coordinate Analysis (PCoA) were calculated with QIIME (Version 1.9.1) and displayed with R software (Version 2.15.3). AMOVA was applied to test the significant differences between groups based on weighted unifrac. Then, the Wilcoxon test was performed to determine significance in alpha diversity indexes across the three dietary groups.

Significant analysis of groups of different classification levels used Metastats analysis with R software, permutation test produce p values, using the Benjamini and Hochberg False Discovery Rate (FDR) method to obtained the q values [23].

### Metagenome Shotgun Sequencing and Bioinformatics Analysis

Three fecal samples from each feeding group were used for metagenome shotgun sequencing, the number of samples were: shoot 5, 6, 7; leaf 3, 5, 6; culm 3, 4, 8. A metagenomic library was prepared using the NEBNext^®^ Ultra™ DNA Library Prep Kit for Illumina (NEB, USA) according to the manufacturer’s protocols. The metagenome libraries were quantified preliminarily using a Qubit^®^ 2.0 Fluorometer (Thermo Scientific). Then, its insert size was determined using an Agilent 2100 Bioanalyzer (Agilent Technologies, Palo Alto, CA, USA). Finally, the effective concentration (> 3 nM) of the library was quantified accurately using real-time PCR. The library preparations were performed on an Illumina Novaseq6000 sequencer (Illumina, San Diego, CA) available at the Novogene Biological Information Technology Co (Beijing, China). Paired-end sequencing reads were filtered by the Readfq (V8) and SoapAligner (soap2.21) function to remove low quality and host reads to acquire clean data for subsequent assembly [24]. A single sample assembly and all samples mixed assembly of clean data analysis was performed using the SOAPdenovo (V2.04) [25]. Open reading frame (ORF) prediction for all scaftigs from the single and mixed assembly groups were performed by MetaGeneMark (V2.10), with a minimum ORF length of 100 bp. For predicted ORFs, the gene catalogues (unigenes) were obtained by removing redundancy, mapping clean data and filtering genes with ≤ 2 reads in each sample. Based on the number and length of the unigenes, we calculated the relative abundance information of each gene in each sample [26, 27]. For functional and taxonomic profiling, we used DIAMOND software (V0.7.9) to blast the unigenes to database with the parameter setting of blast p:–e 1e–5. Databases included: the NCBI NR (Version 20161115), the Kyoto Encyclopedia of Genes and Genomes database (KEGG, Version 201609), the Evolutionary genealogy of genes: Non-supervised Orthologous Groups database (eggnog, Version 4.5), and the Carbohydrate-Active enzymes database (CAZy, Version 20150704). Based on the blast results, we calculated the relative abundance of functional hierarchy in one sample through summing the relative abundance of genes annotated to that functional level [28].The Bray–curtis distance matrix was used to perform a cluster analysis among samples, and the clustering results were integrated with the functional relative abundance of each sample at the first level of CAZy database. Metastats analysis was applied for each functional and pathway gene to acquire a q value.

## Results

### Variation in Gut Microbial Diversity of Captive Giant Pandas by 16S rRNA Gene Sequencing

**Fig. 1:**
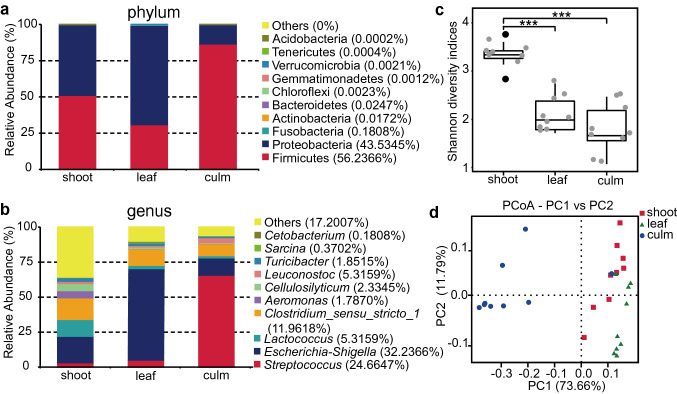
Diversity of the gut microbiomes of the captive giant panda in different groups. (**a**, **b**) Top 10 dominant phyla and genera of gut microbiomes from three groups. “Others” includes bacteria with relative abundance ranked after 10th. The relative abundance for each phylum or genus was the percentage of the number of Tags annotated to a particular phylum or genus to the total number of Tags. (**c**) Comparisons of the Shannon diversity indices in α-diversity of the captive giant panda gut microbiomes among the three dietary groups by Wilcoxon signed-rank test (***P < 0.001). In all panels, the top edge of the box represents the first quartile, and the bottom edge represents the third quartile. The line inside the box represents the median. The gray and black point represents the distribution of sample and outlier respectively. (**d**) A Principal coordinate analysis (PCoA) plot was generated using 16S rRNA data based on weighted unifrac distances for the samples in “shoot”, “leaf” and “culm” groups (*n* = 9 per group). Red square, “shoot” group sample; green triangle, “leaf” group sample; blue point, “culm” group sample

A total of 2,346,890 raw reads were obtained from 27 fecal samples. After quality control, filtering chimaeras and removal of chloroplast sequences, 1,364,526 tags were obtained and grouped into 334 operational taxonomic units (OTUs). The Rarefaction Curves (Fig. S2a) and Rank Abundance Curves (Fig. S2b) showed the observed species number or relative abundance gradually stabilized which suggested the sequencing data were reasonable and that there was uniform species composition within the sample. These results indicated the sample size in this study was sufficient for follow-up analysis. The sequences were assigned into 10 phyla and 160 genera. Firmicutes (161 OTUs, 767,363 Tags, 56.24% of the total of 1,364,526 Tags) and Proteobacteria (101 OTUs, 594,039 Tags, 43.54% of the total Tags) were the two dominant phyla, other phyla showed abundances of 0.22% (72 OTUs, 3,124 Tags, Fig. 1a). In the top 10 genera, *Escherichia-Shigella* (1 OTU, 439,877 Tags, 32.24% of the total Tags) were the predominant genera, followed by *Streptococcus* (12 OTUs, 336,556 Tags, 24.67% of the total Tags), *Clostridium_sensu_stricto_1* (9 OTUs, 163,222 Tags, 11.96% of the total Tags), and *Lactococcus* (6 OTUs, 72,536 Tags, 5.32% of the total tags, Fig. 1b). Differences in the alpha diversity indexes (Shannon and Simpson indexes) in the “shoot” group were higher than the other two groups. (*P*_Shannon_ < 0.001, *P*_Simpson_ < 0.001, Fig. 1c; Table S2). By PCoA analyses, we observed that the “culm” group cluster was separate from the other two groups (Fig. 1d; Table S3).

**Fig. 2:**
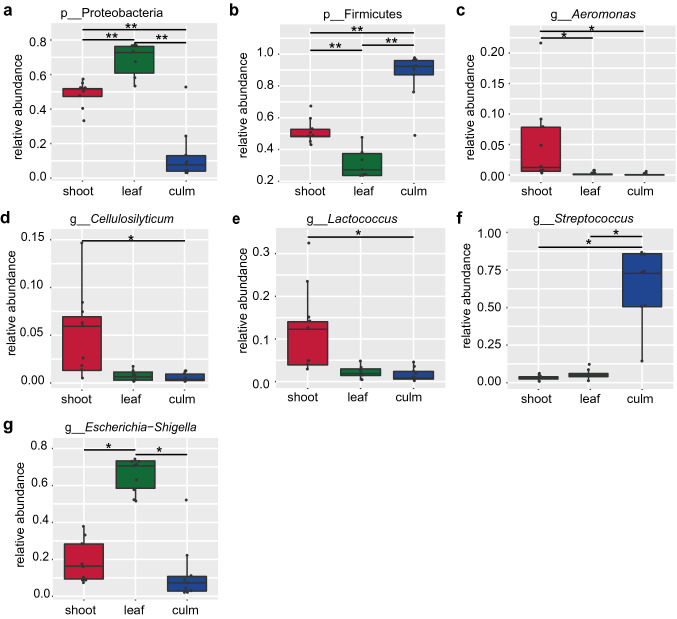
The composition of specific microbiomes at different taxonomic levels in the feces of captive giant pandas from three dietary groups. Panels (**a**–**g**) show details of the significant difference in abundance of microbiomes between the different diet groups by Metastats analysis. Significant differences are determined by q value. Horizontal lines represent the two groups with significant differences, where *q < 0.05; **q < 0.01. In all panels, the top edge of the box represents the first quartile, and the bottom edge represents the third quartile. The line inside the box represents the median. Individual values are shown as dots, and the points outside the box represent the outliers. Relative abundance for each phylum or genus was the percentage of the number of Tags annotated to a particular phylum or genus to the total number of Tags. *p* phylum level, *g* genus level. Red box, “shoot” group; green box, “leaf” group; blue box, “culm” group

An obvious variation on gut microbiome proportion was observed at the phylum (Fig. 1a) and genus levels (Fig. 1b) among the three groups. Further statistical analysis revealed Proteobacteria was highest in the “leaf” group compared with the other two groups (*q* < 0.01, Fig. 2a). However, relative to “leaf” and “shoot” stage, the Firmicutes increased during the “culm” stage (*q* < 0.01, Fig. 2b). At the genus level, *Aeromonas*, *Cellulosilyticum* and *Lactococcus* in the “shoot” group had a markedly higher abundance than the “culm” group (*q* < 0.05, Fig. 2c–e). Whereas, *Streptococcus* were increased significantly in the “culm” group compared with the other two groups (*q* < 0.05, Fig. 2f). In addition, the “leaf” group had the highest *Escherichia-Shigella* among three groups (*q* < 0.05, Fig. 2g).

### Functional Potential of Captive Giant Panda Gut Microbiome by Metagenome Shotgun Sequencing

To investigate the difference in the potential microbiome function of captive giant pandas provisioned with different bamboo parts, we carried out the metagenome shotgun sequencing with nine samples (three per group). In total, 57.5 Gb (5.9 to 6.8 Gb per sample) sequence data were obtained. After assembly, 338.5 Mbp total length scaftigs (N50 = 1660.80 ± 750.99 bp per sample) harboring 500,122 predicted ORFs (≥ 500 bps) were constructed. A gene catalogue consisting of 232,096 genes (unigenes) was annotated and matched against four databases. Finally, 196,370 (84.61%) genes were annotated using the NCBI NR database; 171,890 (74.06%) genes, a total of 4,762 KEGG orthology (KO) groups and 1,575 enzymes (EC) were involved in 253 pathways which were annotated in the KEGG database. A total of 162,154 (69.87%) genes involved in 11,314 orthologous groups (OG) were annotated with the eggNOG database. A total of 3,032 (1.31%) unigenes were involved in 400 enzymes annotated in the Carbohydrate-Active Enzymes (CAZy) database.

### Variation of Predicted Metagenomes of the Giant Panda Gut Microbiome

Annotated by the CAZy database, the “shoot”, “leaf” and “culm” groups completely clustered into three groups (Fig. S3). And higher relative abundance of genes for glycoside hydrolases (GHs) and glycosyl transferases (GTs) were present in the “leaf” and “culm” group compared with the “shoot” group (Fig. S3).

**Fig. 3:**
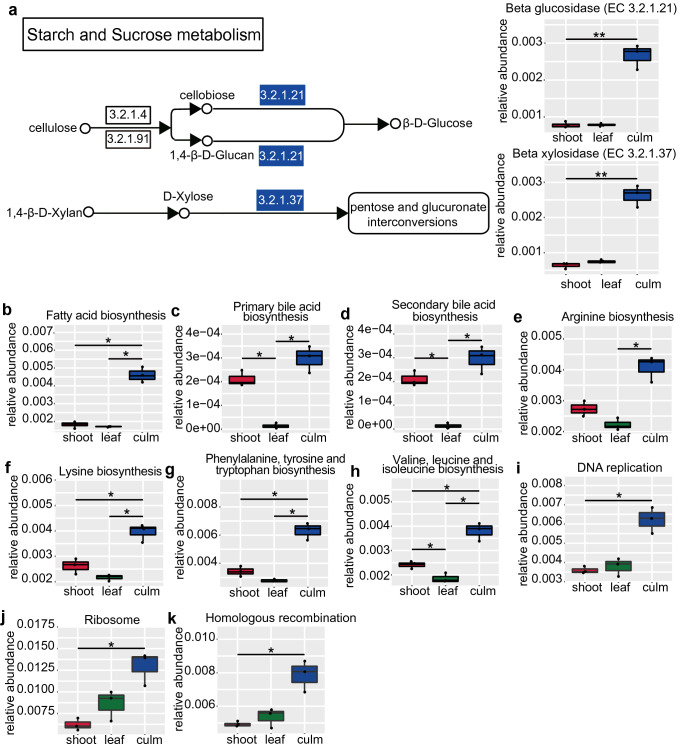
Reinforcement of crude fiber digestion, nutrient biosynthesis and robustness of bacteria related to function in the “culm” group. (**a**) Genes involved in the pathways of cellulose/hemicellulose digestion and utilization. The blue background color shows significantly higher abundance of enzyme genes of gut microbiota in the “culm” group than the other groups. Panels show abundance of genes of nutrient biosynthesis (**b**–**h**) and cell cycle control (**i**–**k**) respectively. Relative abundance for each enzyme was the ratio of the abundance of genes annotated to a particular enzyme to the total abundance of all enzyme genes. The relative abundance of different functional hierarchy (ko) which was equal to the sum of the relative abundance of genes annotated to that functional level. In all panels, the top edge of the box represents the first quartile, and the bottom edge represents the third quartile. The line inside the box represents the median. Individual values are shown as dots. Significant differences are determined by *q* value. Horizontal lines represent two groups with significant differences, where *q < 0.05; **q < 0.01. Red box, “shoot” group; green box, “leaf” group; blue box, “culm” group

Regarding cellulose degradation, only 19 cellulase genes were annotated, but the number of genes was 259 for β-xylosidase (EC 3.2.1.37) and 234 for β-glucosidase (EC 3.2.1.21). Interestingly, the relative abundance of β-xylosidase (EC 3.2.1.37) and β-glucosidase (EC 3.2.1.21) were all significantly enriched in the “culm” group (Fig. 3a, *q* < 0.01). However, the cellulase (EC 3.2.1.4) and cellulase 1,4-β-cellobiosidase (EC 3.2.1.91) in the “culm” group were not differentially represented compared with the other two groups. Furthermore, the “culm” group had higher abundance of the genes for fatty acid biosynthesis (ko00061, Fig. 3b, *q* < 0.05), bile acid transformation (ko00120, ko00121, Fig. 3c and d, *q* < 0.05) and biosynthesis of eight essential amino acids (arginine, ko00220; lysine, ko00300; phenylalanine, tyrosine and tryptophan biosynthesis, ko00400; valine, leucine and isoleucine biosynthesis, ko00290 (Fig. 3e–h, *q* < 0.05) compared with the “leaf” and “shoot” group. In addition, the cell cycle control involved in DNA replication (ko03030), ribosome (ko03010) and homologous recombination genes(ko03440) was over-represented in the “culm” group (Fig. 3i–k).

**Fig. 4:**
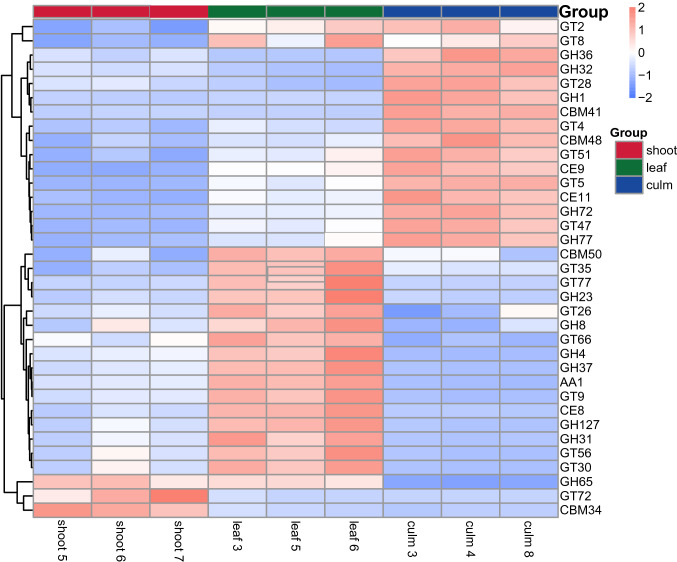
Heatmap of clustering for the relative abundance of CAZy family genes on CAZy level 2. The clustering tree was generated based on the relative abundance of the CAZy family with significant difference by Metastats analysis (*q* value < 0.05). The information of samples and CAZy family annotation were demonstrated along X-axis and Y-axis respectively. The values of the heat map correspond to the Z value of relative abundance for each horizontal line of functionality after normalization. *GH* glucoside hydrolase, *GT* glycosyl transferase, *CBM* carbohydrate-binding module, *CE* carbohydrate esterases, *AA* auxiliary activities. Red rectangle, “shoot” group; green rectangle, “leaf” group; blue rectangle, “culm” group

Interestingly, the “culm” group was enriched in α-amylase families (CBM48 and GH77) [29] that are able to bind and degrade starch. Meanwhile, the relative abundance of CAZy families in the “culm” group, which are involved in the cellulose (GH1) and hemicellulose degrading activity (GH36) [30], and the sucrose (GT4), starch and glycogen (GT5) synthetase activity [31], were significantly higher than the “shoot” and “leaf” groups (*q* < 0.05, Fig. 4).

**Fig. 5:**
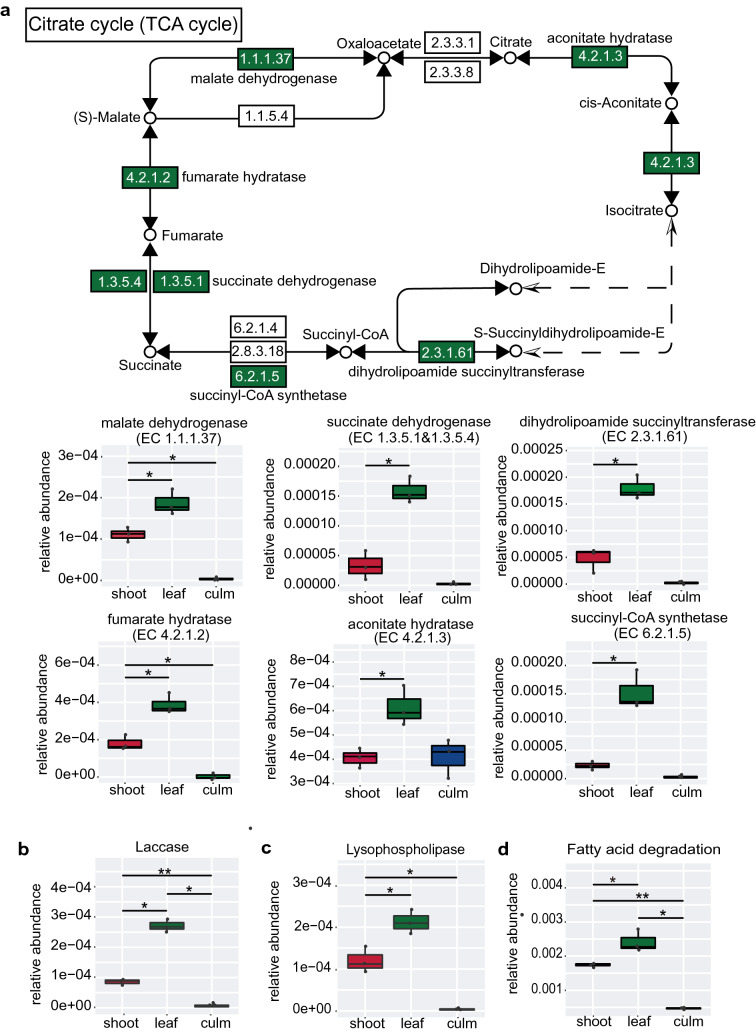
Reinforcement of crude fat digestion, nutrients metabolism and TCA cycle of bacteria related to function in the “leaf” group. The different abundance of enzyme genes of nine fecal samples from giant pandas were annotated in the TCA cycle pathway in the KEGG database (**a**). Green background color shows significantly higher abundance of enzyme genes of gut microbiota in the “leaf” group compared with the other two groups. Panels show details of abundance of the enzyme genes (**a**–**c**) and fatty acid degradation (**d**) respectively. Relative abundance for each enzyme was the ratio of the abundance of genes annotated to a particular enzyme to the total abundance of all enzyme genes. The relative abundance of different functional hierarchy (ko) which was equal to the sum of the relative abundance of genes annotated to that functional level. In all panels, the top edge of the box represents the first quartile, and the bottom edge represents the third quartile. The line inside the box represents the median. Individual values are shown as dots. Significant differences are determined by *q* value. Horizontal lines represent two groups with significant differences, where *q < 0.05; **q < 0.01. Red box, “shoot” group; green box, “leaf” group; blue box, “culm” group

The enzyme genes in the tricarboxylic acid cycle (TCA cycle, ko00020) were significantly more abundant in the “leaf” group than in the “shoot” or “culm” groups (*q* < 0.05, Fig. 5a). We also found 34 genes encoding laccases (EC 1.10.3.2 and EC 1.10.3.-) which were enriched in the “leaf” group (*q* < 0.05, Fig. 5b). The abundance of the lysophospholipase (EC:3.1.1.5) and fatty acid degradation genes (ko00071) were also abundant in the “leaf” group (*q* < 0.05, Fig. 5c and d). In addition, rhodanese (EC 2.8.1.1) and cyanate lyases genes (EC 4.2.1.104), related to cyanide detoxification in the giant panda [11, 32] were enriched in the “shoot” and “leaf” groups respectively (*q* < 0.05, Fig. S4a and b).

## Discussion

Previous research [5] has demonstrated that provisioning a specific part of a single species of bamboo, such as *Bashania fargesii* leaves, *Qiongzhuea opienenss* shoots or *Phyllostachys bissetii* culms, to captive giant pandas may lead to nutritional imbalance, which potentially may have a negative impact on the health of this iconic species. Based on our previous study, we found that the structure and function of the gut microbiome in captive giant pandas significantly changed when consuming a diet consisting of a single bamboo part. The giant panda’s gut microbiota structure in our study is similar to previous investigations [16], but the proportion of the two main phyla, Firmicutes (56.2%) and Proteobacteria (43.5%), are quite different compared to wild giant pandas (Firmicutes > 83% and Proteobacteria > 14%) [10]. The differences of microbiota proportion between wild and captive individuals may be due to several factors related to the captive environment, such as decreased access to a variety of bamboo species, decreased area, increased human disturbances and other variations [6, 33]. Our study revealed that the relative abundance of Firmicutes in the captive giant pandas fed solely a culm diet were the highest among the three groups. Using functional classification in a metagenomic analysis, less cellulase and more hemicellulase related genes were found in the “culm” group. These findings suggest that hemicellulase, but not cellulase, may play an important role in giant panda nutrition, which is consistent with previous studies [11, 12]. Zhu et al. 2011 [10] demonstrated that putative β-glucosidase and xylan 1,4-β-xylosidase genes, which are key enzymes for cellulose and hemicellulose digestion, were found in Firmicutes. Compared with the “leaf” and “shoot” groups, the “culm” group was rich in β-xylosidase and β-glucosidase. However, cellulase was not significantly different between the “culm” and the other two groups. Cellulose is the major constituent of plant cell walls [34]. Cellulose is first cut off by cellulase at the 1,4-β-linkage to form celluldextrin or cellobiose, and then degraded into glucose by β-glucosidase [6, 35]. As with bamboo shoots and leaves, the nutritional source (high crude fiber, low protein and fat) in bamboo culm is poor [5]. Because of this, it may not only be necessary for giant pandas to break down the plant cell walls, but also to further degrade cellobiose and 1, 4-β-D-glucan to produce glucose for energy. Therefore, the “culm” group increased the key enzymes genes for cellulose and hemicellulose digestion and may contribute to the degradation of crude fiber (crude fiber digestibility: “culm” group = 50.54%, “leaf” group = 28.98%, “shoot” group = 18.11%) [5].

Furthermore, the “culm” group contained a high abundance of α-amylase and maltogenic amylase gene families which are related to the degradation of starch [36]. Meanwhile, the genes for fatty acid biosynthesis, and primary and secondary bile acid biosynthesis were also enriched in the “culm” group. Bile acids have the ability to promote digestion and absorption by emulsifying liposoluble nutrients [37]. Consistently, our previous study showed that the “culm” group had the highest crude fat digestibility (“culm” group = 77.08%, “leaf” group = 41.43%, “shoot” group = 57.84%) [5]. Thus, the digestible energy for the giant panda may increase during the nutrient-deficient “culm” stage (“culm” group = 69.37 MJ/d, “leaf” group = 35.28 MJ/d, “shoot” group = 14.80 MJ/d) [5] by improving crude fiber, starch and fat digestion with the help of gut microbiota. In addition, the genes encoding for microbiome cell cycle control and the biosynthesis of eight essential amino acids were higher in the “culm” group than both the “leaf” and “shoot” groups. Thus, the gut microbiota may enhance their cell cycle control within the giant panda digestive system to guarantee related bacteria species responsible for nutrient digestion. It is likely that giant pandas undergo microbiome changes to generate more enzymes to digest the least digestible culms, as a means to compensate for the poor nutrition provided by culms. It is noticeable that the “culm” group absorbed the highest amounts of calories and fiber, however was in short energy supply with less TCA cycle activity. Therefore, it can be speculated that digestion of fiber requires energy input and yields low caloric extraction from the culm so that the pandas lost weight during this period [5].

The relative abundance of Proteobacteria in the “leaf” group was significantly higher than the other two groups. Previous studies have demonstrated that Proteobacteria is mainly associated with energy accumulation in mammals, such as humans, mice and black howler monkeys [38–40]. The gut microbiota of giant pandas have genes encoding for laccase [41], but more recent studies showed only low abundance of putative laccase genes in giant panda metagenomes [11, 12]. In this study, we also found a small number of the laccase gene in captive giant pandas, however the “leaf” group had a higher abundance of laccase genes than the other two groups. Laccase depolymerizes the hemicellulose bound by lignin [7], which may benefit the digestion of hemicellulose from bamboo leaves. In our previous study, the digestible crude fat intake by giant pandas in the “leaf group” was highest (“leaf” group = 53.55 g/d, “culm” group = 25.97 g/d; “shoot” group = 31.77 g/d) [5]. Considering the lipase activity of the giant panda is much lower than that in the brown bear [3], the hindgut microbiota may use the remaining crude fat which the foregut does not digest. Interestingly, the “leaf” group had the highest level of genes for lysophospholipase and fatty acid degradation. Moreover, the “leaf” group had the highest level of the enzyme gene for the TCA cycle. The excess fatty acids not utilized by the host might be completely oxidized eventually through TCA cycle [42]. These results suggest that microbiota probably play a key role in fat digestion, and fat is a primary energy source when giant pandas eat fat-enriched bamboo leaves.

During their long evolutionary history, mammals and their indigenous microbial communities have co-evolved [43]. Our results show that the gut microbiota of giant pandas can help digest bamboo. In addition, the gut microbiome of the giant panda contained rhodanese and cyanate lyases which might play a role in cyanide detoxification to combat the toxicity of cyanide which is an abundant secondary metabolite in bamboo [44]. Rhodanese was also found in the gut microbiome of other bamboo-eating animals (e.g., red pandas) [15]. Therefore, these results indicate that the gut microbial structure and function changed when provisioned with a single bamboo part and may be an important adaptive mechanism for giant pandas. Other species that feed on bamboo such as the bamboo lemur and the red panda also have gut microbiota adapted to a bamboo diet [32, 45]. The composition and potential function of the gut microbiome from giant pandas and other bamboo eating animals is mainly shaped by diet type and diet diversity [46, 47]. Nevertheless, extensive studies have shown that gut microbes are closely related to the host's nutrition and health status [48, 49]. Compared with captive pandas, in the wild, giant pandas have access to, and forge on, more diverse species of bamboo and therefore consume different bamboo parts during different seasons [6, 13]. Whether the long-term feeding of a single bamboo species and bamboo part affects the health and digestive mechanism of captive giant pandas requires further investigation.

While this study discovered subtle gut microbiome adaptations of captive giant pandas to different bamboo diets, proving the adaptability of microbes to diet, there are some limitations. One limitation being that the sample size of the metagenome was too small. The selection of the metagenomic samples were based on the results of 16S rRNA gene sequencing. From each group of nine samples, three samples were selected according to the bacterial abundance that best represented the bacterial composition characteristics of the group, and the bacterial abundance in the selected metagenomic samples were consistent with the 16S results. In future studies, a larger sample size should be used for verification.

In conclusion, our 16S rRNA and metagenomic gene data show that the gut microbiome communities and function of captive giant pandas change markedly with the different nutritional levels of the three selected bamboo diets. In the high fiber and poor nutritional “culm” group, microbiomes for digestive enzymes genes of crude fiber, sugar and crude fat were high. Thus, giant pandas undergo microbiome changes to generate more enzymes to digest the least digestible culms to compensate for the poor nutrition provided by culms. In the fat-abundant “leaf” group, the over-represented genes for the fatty acid metabolism and TCA cycle pathway suggest that microbiota may play a key role in fat digestion. Furthermore, fat may be used as a primary energy source when giant pandas with low lipase activity eat fatty bamboo leaves. Our findings indicate that the gut microbiome plays an essential role in nutrient digestion in the giant panda, and is able to adapt to different bamboo diets.

## Supplementary Information

Below is the link to the electronic supplementary material.

Supplementary File1 (DOCX 671 kb)

## Data Availability

Raw data can be obtained from the Sequence Read Archive, under BioProject PRJNA433781.
