# Supplementary material for: Consuming Different Structural Parts of Bamboo Induce Gut Microbiome Changes in Captive Giant Pandas
Source: Curr Microbiol. 2021 Jun 9;78(8):2998–3009. doi: 10.1007/s00284-021-02503-y (PMC8289812; doi:10.1007/s00284-021-02503-y)
Supplement: Supplementary file 1 — Supplementary File1 (DOCX 671 kb) [file 284_2021_2503_MOESM1_ESM.docx]

## Supplementary Files

## Supplemental Figures
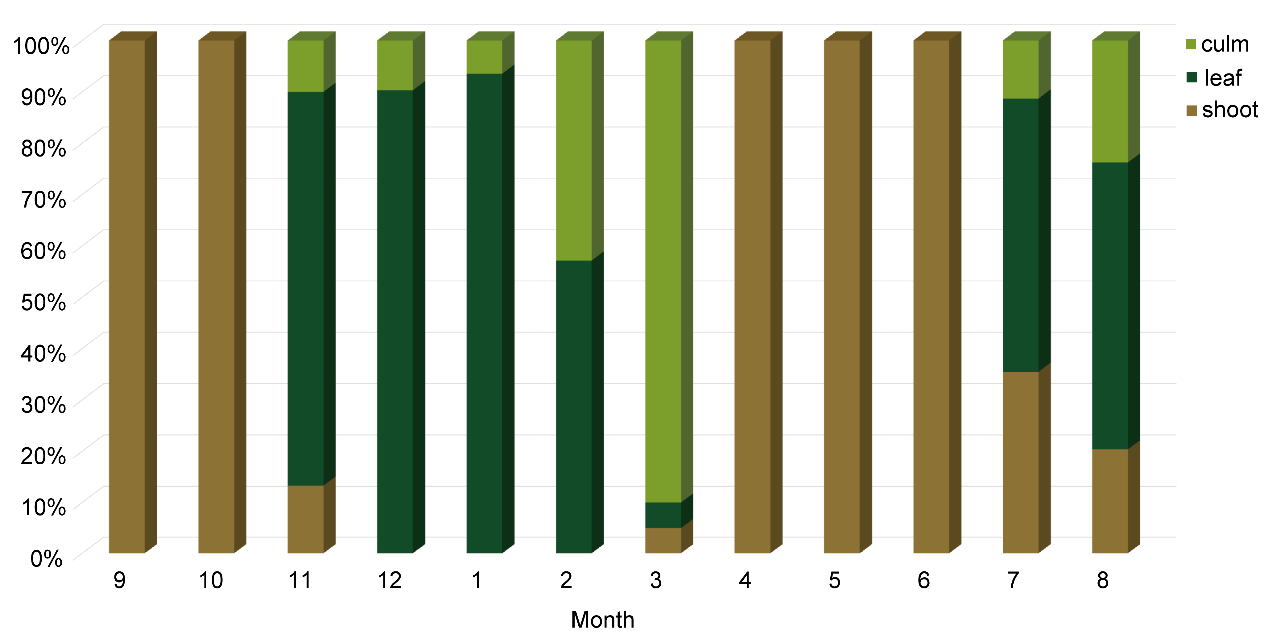


**Fig. S1** The feeding of bamboo shoot, leaf and culm by giant pandas in captivity during the 1-year trial period (
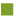
,bamboo culm diet;
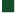
bamboo leaf diet;
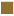
,bamboo shoot diet).


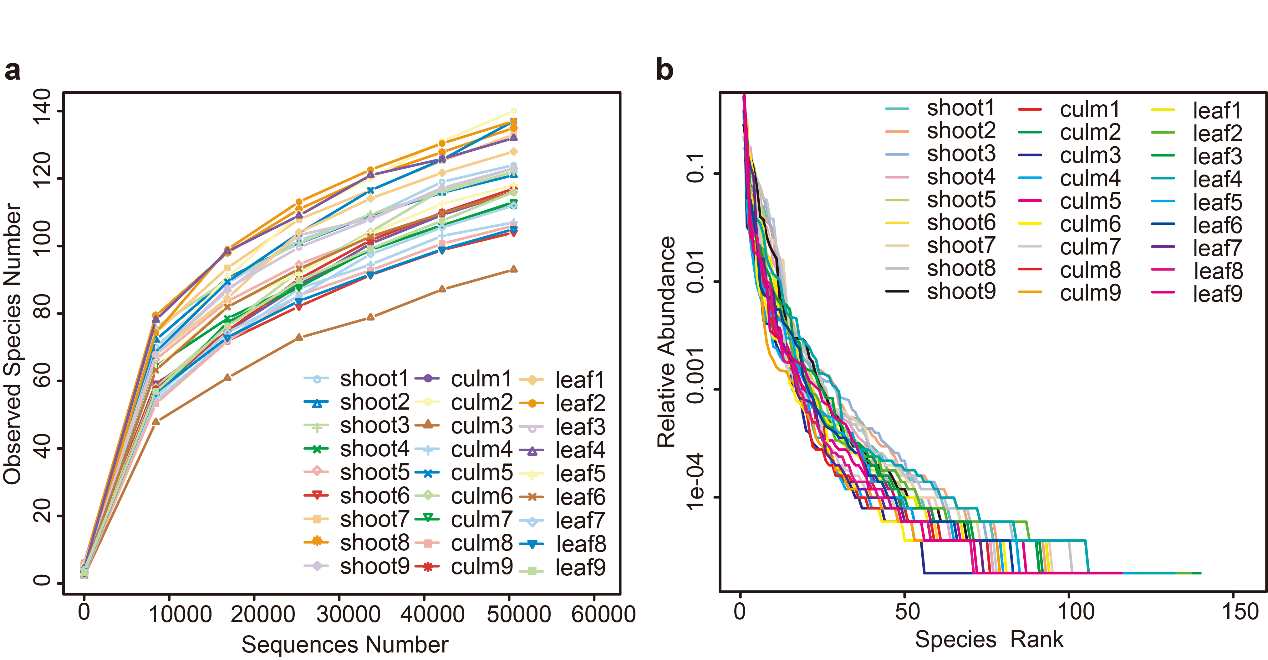


**Fig. S2** Giant panda fecal sample Rarefaction Curve (a) and Rank Abundance Curve (b)

***
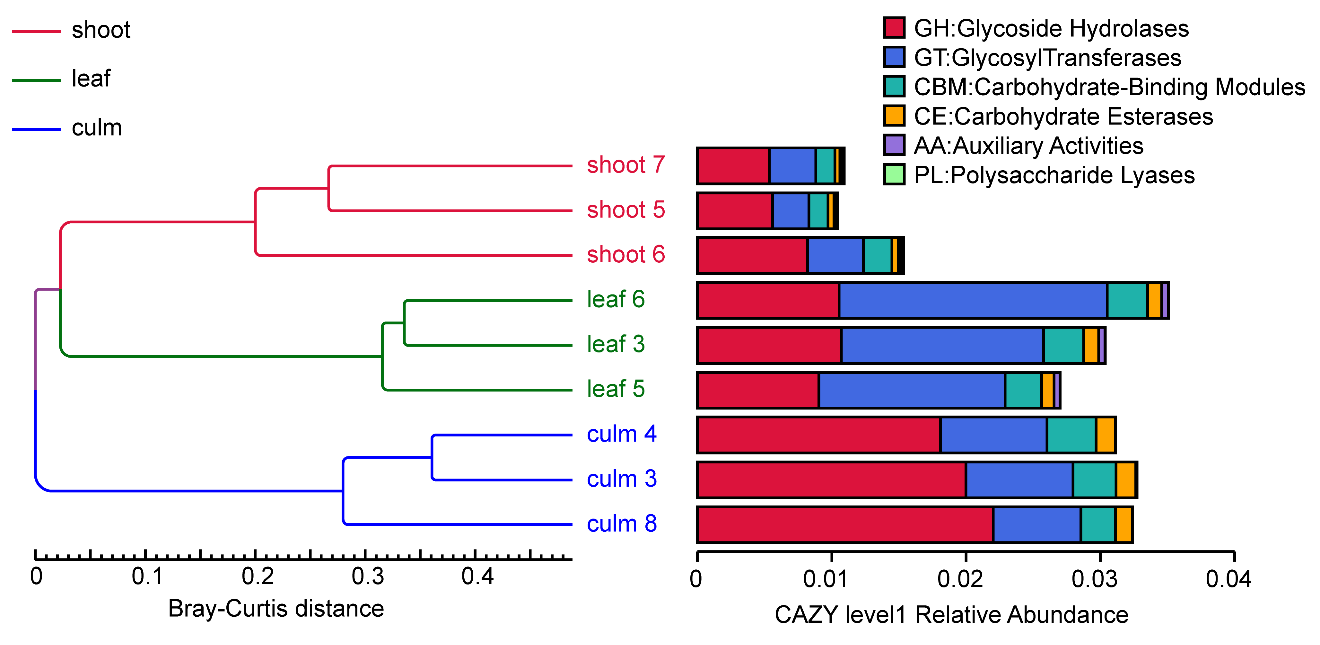
***

**Fig. S3** The difference of gut microbiomes in CAZy database by feeding different bamboo diets. Hierarchical clustering based on the Bray–Curtis distance similarity for fecal samples in the left. Red line, “shoot” group; green line, “leaf” group; blue line: “culm” group; The relative abundance of CAZyme in the “shoot”, “leaf” and “culm” groups in the right. Relative abundance for each CAZyme was the ratio of the abundance of genes annotated to a particular CAZyme to the sum of all CAZyme genes abundance. CAZy functional level 1 are indicated by tip label colors. CAZyme were annotated based on metagenome shotgun sequencing of 9 fecal samples of giant pandas (n = 3 per group).


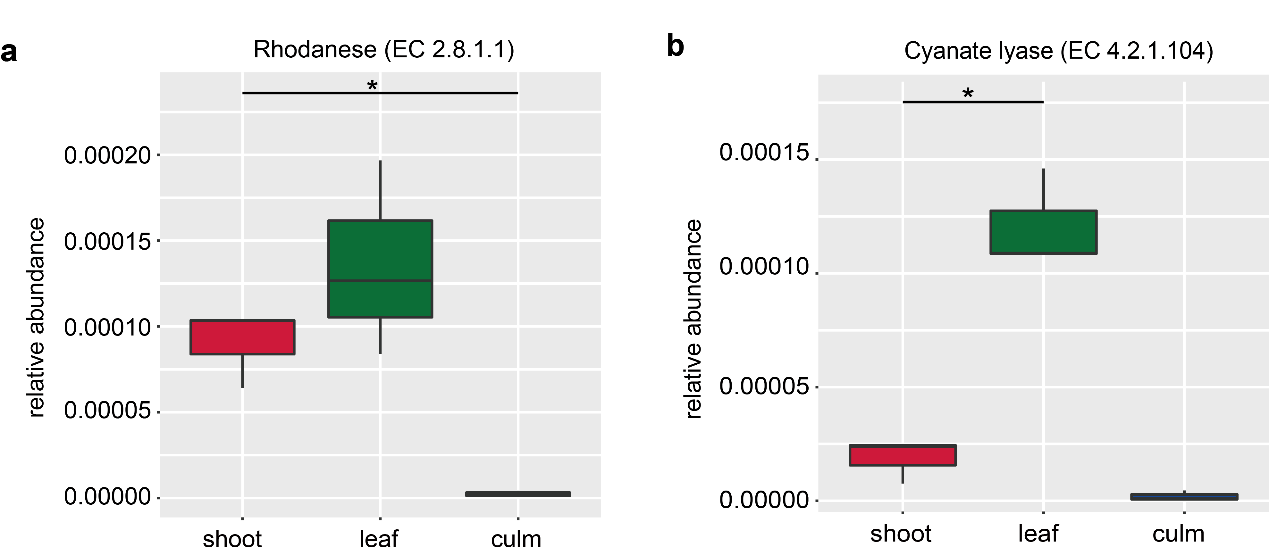


**Fig. S4** Relative abundance of the rhodanese (a) and cyanate lyase (b) in fecal sample from captive giant panda. Relative abundance for each enzyme was the ratio of the abundance of genes annotated to a particular enzyme to the total abundance of all enzyme genes. In all panels, the top edge of the box represents the first quartile, and the bottom edge represents the third quartile. The line inside the box represents the median. Significant differences are determined by q value. Horizontal lines represent two groups with significant differences, where *q < 0.05. Red box, “shoot” group; green box, “leaf” group; blue box, “culm” group.

## Supplemental Table

**Table S1.** Sample collection

| Group | Sample number | Sampling date | Studbook No. | Name | Sex | Date of Birth |
| --- | --- | --- | --- | --- | --- | --- |
| shoot | shoot1 | 2016-10-12 | 574 | QIU BANG | M | 2003-9-8 |
|  | shoot2 | 2016-10-13 | 614 | XING BANG | M | 2005-8-23 |
|  | shoot3 | 2016-10-13 | 680 | XING RONG | F | 2007-8-13 |
|  | shoot4 | 2016-10-14 | 665 | XIANG BING | F | 2007-6-30 |
|  | shoot5 | 2016-10-14 | 717 | SHU XIANG | M | 2008.8.4 |
|  | shoot6 | 2016-10-14 | 731 | XI LAN | M | 2008.8.30 |
|  | shoot7 | 2010-10-17 | 663 | AI BANG | F | 2006.12.23 |
|  | shoot8 | 2016-10-19 | 573 | LONG BANG | M | 2003-9-8 |
|  | shoot9 | 2016-10-19 | 624 | QIAO QIAO | M | 2004 |
| leaf | leaf1 | 2016-11-10 | 532 | FU FU | M | 2001-8-25 |
|  | leaf2 | 2016-11-11 | 624 | QIAO QIAO | M | 2004 |
|  | leaf3 | 2016-11-24 | 663 | AI BANG | F | 2006-12-23 |
|  | leaf4 | 2017-01-06 | 480 | SHU QING | F | 1999-8-3 |
|  | leaf5 | 2017-01-09 | 738 | YONG BANG | M | 2008-9-13 |
|  | leaf6 | 2017-01-10 | 711 | GONG ZAI | M | 2008-7-26 |
|  | leaf7 | 2017-01-11 | 574 | QIU BANG | M | 2003-9-8 |
|  | leaf8 | 2017-01-18 | 584 | YONGYONG | M | 2004-8-26 |
|  | leaf9 | 2017-01-20 | 680 | XING RONG | F | 2007-08-13 |
| culm | culm1 | 2017-03-15 | 554 | YA SHUANG | F | 2002-08-28 |
|  | culm2 | 2017-03-16 | 574 | QIU BANG | M | 2003-09-08 |
|  | culm3 | 2017-03-21 | 614 | XING BANG | M | 2005-08-23 |
|  | culm4 | 2017-03-22 | 652 | MENGMENG | F | 2006-09-13 |
|  | culm5 | 2017-03-22 | 663 | AI BANG | F | 2006-12-23 |
|  | culm6 | 2017-03-22 | 765 | BEI CHUAN | F | ~2006+/-2yr |
|  | culm7 | 2017-03-28 | 680 | XING RONG | F | 2007-8-13 |
|  | culm8 | 2017-03-28 | 593 | SI YUAN | F | 2004-10-22 |
|  | culm9 | 2017-03-29 | 540 | XIONGBANG | M | 2001-12-17 |

**Table S2.** Alpha-diversity of gut microbiota in the fecal samples from captive giant panda

| item | shoot | culm | leaf | SEM | *P* value | | |
| --- | --- | --- | --- | --- | --- | --- | --- |
|  |  |  |  |  | culm  vs  leaf | culm vs shoot | leaf  vs shoot |
| observed_species | 121.125 | 116.889 | 120.778 | 2.218 | 0.588 | 0.5126 | 0.9089 |
| shannon | 3.316 | 1.762 | 2.08 | 0.151 | 0.06 | <0.001 | <0.001 |
| simpson | 0.844 | 0.488 | 0.541 | 0.038 | 0.3476 | <0.001 | <0.001 |
| chao1 | 152.44 | 171.979 | 153.083 | 5.282 | 0.9319 | 0.6091 | 0.5512 |
| ACE | 160.988 | 168.031 | 160.281 | 3.513 | 0.458 | 0.4245 | 0.9542 |
| goods_coverage | 0.99922 | 0.99916 | 0.99919 | 0.00003 | 0.665 | 0.315 | 0.636 |
| PD_whole_tree | 9.923 | 10.028 | 9.849 | 0.142 | 0.5706 | 0.9094 | 0.6497 |

SEM, standard error of the mean. The Wilcoxon test was used to test for the alpha diversity indexes across the three dietary groups. *P*-value less than 0.05 indicates a significant difference inter-group.

**Table S3.** Intergroup difference analysis by AMOVA

| vs_group | SS | df | MS | Fs | *P*-value |
| --- | --- | --- | --- | --- | --- |
| culm vs shoot | 0.548358(0.287059) | 1(16) | 0.548358(0.0179412) | 30.5642 | <0.001 |
| culm vs leaf | 0.672705(0.244953) | 1(16) | 0.672705(0.0153096) | 43.9402 | <0.001 |
| leaf vs shoot | 0.0661787(0.14185) | 1(16) | 0.0661787(0.00886564) | 7.46463 | 0.001 |
| culm vs leaf vs shoot | 0.858161(0.336931) | 2(24) | 0.42908(0.0140388) | 30.5639 | <0.001 |

Note: SS: Sum of squares; df: degree of freedom；MS: mean square (SS/ df); Fs: F standard value. *P*-value less than 0.05 indicates a significant difference inter-group. The value of the residuals was shown in parentheses.
